# Cardiovascular Benefits of Wearing Particulate-Filtering Respirators: A Randomized Crossover Trial

**DOI:** 10.1289/EHP73

**Published:** 2016-08-26

**Authors:** Jingjin Shi, Zhijing Lin, Renjie Chen, Cuicui Wang, Changyuan Yang, Jing Cai, Jingyu Lin, Xiaohui Xu, Jennifer A. Ross, Zhuohui Zhao, Haidong Kan

**Affiliations:** 1 School of Public Health, Key Lab of Public Health Safety of the Ministry of Education and Key Lab of Health Technology Assessment of the Ministry of Health,; 2 Shanghai Key Laboratory of Atmospheric Particle Pollution and Prevention (LAP^3^), and; 3 Zhongshan Hospital, Fudan University, Shanghai, China; 4 Department of Epidemiology and Biostatistics, and; 5 Department of Public Health Studies, Texas A&M School of Public Health, College Station, Texas, USA

## Abstract

**Background:**

Practical approaches to protect individuals from ambient particulate matter (PM) are urgently needed in developing countries. Evidence on the health benefits of wearing particulate-filtering respirators is limited.

**Objectives:**

We evaluated the short-term cardiovascular health effects of wearing respirators in China.

**Methods:**

A randomized crossover trial was performed in 24 healthy young adults in Shanghai, China in 2014. The subjects were randomized into two groups and wore particulate-filtering respirators for 48 hr alternating with a 3-week washout interval. Heart rate variability (HRV) and ambulatory blood pressure (BP) were continuously monitored during the 2nd 24 hr in each intervention. Circulating biomarkers were measured at the end of each intervention. Linear mixed-effect models were applied to evaluate the effects of wearing respirators on health outcomes.

**Results:**

During the intervention periods, the mean daily average concentration of PM with an aerodynamic diameter < 2.5 μm (PM2.5) was 74.2 μg/m3. Compared with the absence of respirators, wearing respirators was associated with a decrease of 2.7 mmHg [95% confidence interval (CI): 0.1, 5.2 mmHg] in systolic BP and increases of HRV parameters, including 12.5% (95% CI: 3.8%, 21.2%) in high frequency (HF) power, 10.9% (95% CI: 1.8%, 20.0%) in the root mean square of the successive differences, and 22.1% (95% CI: 3.6%, 40.7%) in the percentage of normal RR intervals with duration > 50 msec different from the previous normal RR interval (pNN50). The presence of respirators was also associated with a decrease of 7.8% (95% CI: 3.5%, 12.1%) in the ratio of low frequency (LF)/HF power.

**Conclusions:**

Short-term wearing of particulate-filtering respirators may produce cardiovascular benefits by improving autonomic nervous function and reducing BP.

**Citation:**

Shi J, Lin Z, Chen R, Wang C, Yang C, Cai J, Lin J, Xu X, Ross JA, Zhao Z, Kan H. 2017. Cardiovascular benefits of wearing particulate-filtering respirators: a randomized crossover trial. Environ Health Perspect 125:175–180; http://dx.doi.org/10.1289/EHP73

## Introduction

Cardiovascular health hazards are among the primary health risks associated with air pollution exposure (Donaldson et al. 2013). A number of population-based epidemiological studies have demonstrated that short-term exposure to air particulate matter (PM) is associated with reduced heart rate variability (HRV) (Baccarelli et al. 2008; Ren et al. 2010) and increased blood pressure (Hampel et al. 2011) and inflammation levels (Liu et al. 2009), all of which can act as indicators for potential adverse cardiovascular health effects. PM with an aerodynamic diameter < 2.5 μm (PM_2.5_) is particularly associated with cardiovascular damage and can act as a stimulus to trigger local cytokine production and systemic inflammation (Schins et al. 2004).

In developing countries where the population size is large and air pollution levels are high, the disease burden associated with PM_2.5_ is more severe than that in North America and Europe. The Global Burden of Disease (GBD) study estimated that ambient PM ranked fourth among all risk factors in China in 2010, contributing nearly 8% of the total disability-adjusted life years (Yang et al. 2013). Given that it is not easy to cut the emission of air pollutants in a short time in developing countries, approaches that can reduce individual exposure are considered to be practical and cost-effective in protecting the public from ambient PM. These approaches are urgently needed in highly polluted countries such as China and India. Previous studies have reported the health benefits associated with the use of indoor air purifiers and oral supplements (Chen et al. 2015a; Romieu et al. 2008). Wearing particulate-filtering respirators, one of the most convenient and affordable protective measures, is becoming increasingly popular in China, particularly in outdoor environments. However, evidence of their health benefits is limited (Langrish et al. 2009, 2012).

Therefore, we designed a randomized controlled crossover trial to evaluate the potential cardiovascular benefits associated with wearing a particulate-filtering respirator in a group of healthy young adults in Shanghai, China. We examined blood pressure (BP), HRV, and circulating biomarkers of important pathways by which PM exposure leads to adverse cardiovascular outcomes (Brook et al. 2010).

## Materials and Methods

### Study Design and Participants

We conducted a randomized crossover trial in a group of healthy college students at Fudan University, Shanghai, China, during the period from 21 March to 13 April 2014. The entire study was completed within 1 month to avoid potential confounders resulting from long-term and seasonal trends of health outcomes.

Initially, we recruited 30 students with no history of tobacco smoking (never smokers) or alcohol addiction, no clinically diagnosed chronic cardiopulmonary diseases (including asthma, rhinitis, and others) and no recent infections. Because all participants lived in campus dormitory rooms (a typical dormitory room is shared by four or six persons) and studied within the campus, they were seldom exposed to environmental tobacco smoke because smoking was banned in all public places on the campus.

The participants were equally randomized into two groups and wore particulate-filtering respirators for 48 hr alternating with a 3-week washout interval. Specifically, in the first intervention period, one group wore the designated high-efficiency particulate-filtering respirators for 48 hr as the intervention group, while the other group behaved as usual (the control group). After a 3-week wash-out interval, the two groups exchanged roles and completed the second intervention period. HRV and ambulatory BP were continuously monitored during the second 24 hr of each intervention. Blood samples were collected at the end of each intervention period at approximately the same time to measure circulating biomarkers of inflammation, vasoconstriction, and coagulation.

Participants were instructed on how to wear the respirators so that they fit with the participants’ faces closely and comfortably. Participants were required to wear the respirators in the standard position every time. During the interventions, they acted as usual but were required to take a 1-hr walk along the road outside the campus to simulate a regular traffic exposure pattern. The intervention group was required to wear their respirators for all the time they were outdoors and as much as possible when they were indoors. The subjects were asked to record their feelings about the fit of the respirator and their comfort when using it (on a scale from 0 to 10 referring to the worst fit/comfort to the best), the duration of wearing the respirator (percentage of wearing time both indoors and outdoors), and their health (headache, dizziness, tightness of breath, tiredness, etc.) using a standardized questionnaire twice per day (i.e., 0900 and 1500 hours). The demographic characteristics including age, standing height (cm), and weight (kg) were recorded at baseline.

This study was approved by the Institutional Review Board of the School of Public Health, Fudan University (IRB number 2014-01-0473). Written informed consent was obtained from all participants before enrollment, and the study was registered with and approved by ClinicalTrials.gov with the identifier NCT02238028.

### Respirators

Disposable particulate respirators (8210V; 3M™) were used in this study. These respirators are capable of filtering ≥ 95% of 0.3-μm nonoil particulates, meeting National Institute for Occupational Safety and Health (NIOSH) N95 standards (Kang et al. 2011). An expiration valve was installed in the respirators. Qualitative respirator fit testing on the face-to-respirator seal was performed before the intervention study using the 3M™ Qualitative Fit Test Apparatus FT-30 (3M™, USA). First, the subjects positioned the respirators and then placed the professional testing hood on their heads. A bitter-tasting agent was sprayed into the hood. If the subject did not taste the bitter agent at all, the respirators were worn correctly. This testing was repeated three times requiring the subjects to perform three different movements, including standing and slightly turning their head left and right, standing and half crouching, and standing and reading. No detection of bitter taste in any test was considered as formally accepted.

### Heart Rate Variability

A 12-lead continuous electrographic Holter monitor (Seer Light, GE Medical Systems) was installed in each participant during the second 24-hr period of each intervention (from 0800 to 0800 hours). A total of eight parameters of HRV were analyzed including four time-domain indices and four frequency-domain indices. The time-domain variables included *a*) the standard deviation of the normal-to-normal interval (SDNN), estimating the overall HRV; *b*) the root mean square of the successive differences (rMSSD), estimating the short-term components of HRV and a sensitive indicator of vagal tone; *c*) the standard deviation of the average NN intervals calculated over short periods (SDANN); and *d*) the percentage of normal RR intervals with duration > 50 msec different from the previous normal RR interval (pNN50). The frequency-domain parameters included high frequency (HF) power (0.15–0.4 Hz), low frequency (LF) power (0.04–0.15 Hz), very low frequency (VLF) power (0.01–0.04 Hz), and the ratio of LF to HF (LF/HF). The average heart rate and HRV parameters were analyzed using a MARS Holter system (GE Healthcare); the analyses were performed by professional clinical technologists who were blinded to the study design.

### Ambulatory Blood Pressure

A portable, noninvasive, automated ambulatory BP monitoring and recording instrument (Model 90217, Spacelabs) was installed on each subject during the second 24-hr period of each intervention (from 0800 to 0800 hours). The instrument was placed over the left brachial artery, and BP was measured every 15 min during the day (0600–2200 hours) and every 30 min at night (2200–0600 hours). During the measurement with a pumping signal, the subjects were required to refrain from moving until the pump stopped. At least 60 measurements (out of the total 80 measurements) were considered effective monitoring of the BP.

### Circulating Biomarkers

At the end of each intervention, participants were asked to rest in a quiet room for half an hour. Peripheral venous blood samples were collected and centrifuged immediately. The sera were collected and stored at –80°C within 30 min to minimize the *in vitro* changes in biomarker proteins. Five circulating biomarkers were selected for quantitative analyses because they were all significantly associated with PM_2.5_ in our previous studies (Chen et al. 2015a, 2015b). These biomarkers included endothelin-1 (ET-1), P-selectin, vascular cell adhesion molecule-1 (VCAM-1), fibrinogen, and von Willebrand factor (vWF). VCAM-1, fibrinogen, P-selectin, and vWF were measured using the MILLIPLEX® MAP human cytokine/chemokine kit (EMD Millipore Corporation), and ET-1 was measured using enzyme-linked immunosorbent assays (ELISAs). All tests were performed according to the manufacturer’s instructions.

### Environmental Data

The PM_2.5_ levels were continuously monitored both indoors and outdoors simultaneously throughout the study period using a direct-reading personal aerosol monitor (SidePak AM510; TSI) based on the light-scattering method. For the indoor environment, we measured PM_2.5_ concentrations in 2 men’s dormitory rooms and 2 women’s dormitory rooms. These rooms were randomly selected from a total of 10 rooms (5 men’s rooms and 5 women’s rooms). For the outdoor environment, monitors were installed on the rooftop of the men’s dormitory building. All of the dormitory rooms were located within 50 m of each other. Before the whole project began, all devices were calibrated with a nearby national monitoring station by placing them within 20 m of the sampling inlet of the monitoring station. The station used the classical tapered element oscillating microbalance (TEOM) method to measure the ambient PM_2.5_. In addition, indoor and outdoor temperature and relative humidity were continuously monitored and recorded by a HOBO® data logger (Onset Computer Corporation).

### Data Analyses

We used paired Student’s *t*-tests to compare the health indicators in the absence and presence of respirators. HRV and blood biomarker data were log_10_-transformed before regression analyses because of the approximate log-normal distribution. To account for the repeated measurements of health outcomes, we applied linear mixed-effect models to investigate the effects of wearing respirators (Chen et al. 2015a). This model allowed each subject to serve as his or her own control over time, accounting for the between-subject variations that did not change over time. The intervention was coded as a dummy variable (i.e., 1 for wearing the respirator and 0 for not wearing the respirator) and was analyzed as a fixed-effect term in the model. Age, sex, body mass index, PM_2.5_ concentration, 48-hr mean temperature and 48-hr mean humidity were introduced into the model as fixed-effect terms. Finally, we incorporated random-effect intercepts for subjects to account for correlations between repeated measurements.

The estimates for HRV parameters and circulating biomarkers are presented as the average percentage changes and their 95% confidence intervals (CIs). The estimates for BP are presented as the average absolute changes and 95% CIs. All statistical tests were two-sided with an alpha of 0.05. All analyses were performed using the “lme4” package for R software (version 2.15.3; R Project for Statistical Computing).

## Results

### Descriptive Statistics

**Figure 1:**
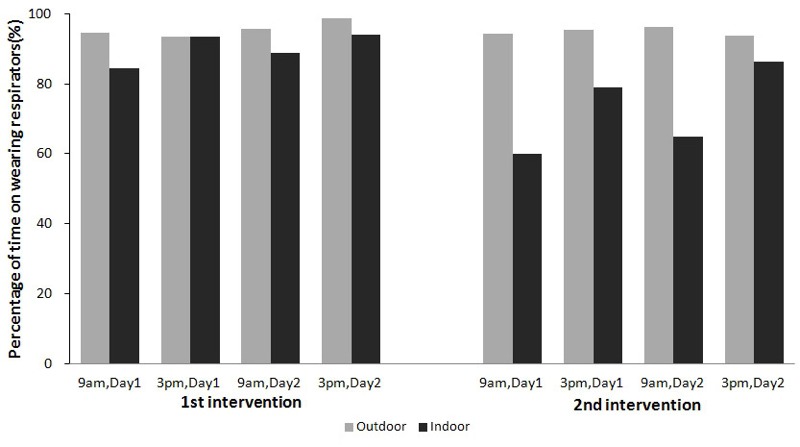
Proportion of time spent wearing respirators outdoors and indoors.

Six participants dropped out of the study in the middle because of sickness (*n* = 2), moving to another campus (*n* = 2), and personal reasons (*n* = 2). Therefore, 24 subjects completed the two periods of intervention. The average age of the 24 subjects was 23 years; males accounted for 54.2% of the total number of participants, and the overall average body mass index was 22 ± 4 kg/m^2^. The scores for respirator fit and participant comfort during the study period were 6 and 5 on average, respectively, suggesting acceptable toleration of wearing the respirators. On average, the participants wore their respirators for > 90% of their time outdoors and 82% of their time indoors, indicating good compliance with the intervention (Figure 1).

The mean daily average concentrations of PM_2.5_ were 74.2 μg/m^3^ outdoors and 85.2 μg/m^3^ indoors during the intervention period (Table 1), more than three times higher than the WHO guideline for a daily average (25 μg/m^3^) (Zhang et al. 2014). The concentrations of indoor PM_2.5_ were slightly higher than those of outdoor PM_2.5_, but the difference was not statistically significant. Considering the particulate-filtration efficiency of the respirator and the proportion of wearing time, the estimated time-weighted exposure levels of PM_2.5_ for subjects wearing respirators were 7.1 μg/m^3^ outdoors and 19.3 μg/m^3^ indoors on average. During the study period, the mean outdoor temperature and relative humidity were 12.9°C and 61%, respectively.

**Table 1:** Air pollutant concentrations and weather conditions (daily 24-hr means, 4 days) during the intervention periods.

Air pollutants	Mean	SD	Min	P25	Median	P75	Max
Outdoor
PM_2.5_ (μg/m^3^)	74.2	38.3	32.0	51.0	61.5	84.0	227.0
NO_2_ (μg/m^3^)	49.1	23.8	16.6	30.1	42.3	62.3	107.6
SO_2_ (μg/m^3^)	11.1	5.0	5.2	6.9	10.3	13.5	32.2
CO (mg/m^3^)	0.4	0.2	0.1	0.2	0.3	0.4	1.3
Temperature (°C)	12.9	4.6	6.4	10.0	13.1	14.7	21.9
Humidity (%)	61.0	17.4	43.0	44.3	57.0	80.3	83.0
Indoor
PM_2.5_ (μg/m^3^)	85.2	43.6	26.7	54.0	73.0	100.3	240.7
Temperature (°C)	20.9	1.4	15.5	20.0	21.0	22.2	25.9
Humidity (%)	47.6	7.8	27.4	41.2	48.9	51.5	78.3
Notes: CO, carbon monoxide; NO_2_, nitrogen dioxide; P25, 1st interquartile value of PM_2.5_ concentration; P75, 3rd interquartile value of PM_2.5_ concentration; PM_2.5_, particulate matter with aerodynamic diameter ≤ 2.5 μm; SD, standard deviation; SO_2_, sulfur dioxide. Outdoor PM_2.5_ measurements were performed over 4 days, and the indoor environment was monitored over 4 days in four dormitory rooms.							

### Effects of Wearing Respirators

Subjects who wore respirators had higher levels of most HRV parameters and lower levels of BP and circulating biomarkers than those who did not wear respirators (Table 2). The differences for HF power and pNN50% reached statistical significance. The LF/HF was significantly higher in the absence of respirators.

**Table 2:** Comparisons (means and standard deviations) of various cardiovascular outcomes in participants wearing respirators or not during the intervention periods.

Parameters	Respirator (*n* = 24)	No respirator (*n* = 24)	*p*
Blood pressure
SBP (mmHg)	107.3 ± 8.0	109.0 ± 7.4	0.097
DBP (mmHg)	70.0 ± 5.0	70.8 ± 4.8	0.235
Heart rate variability
LF power (msec^2^)	899.4 ± 601.3	838.5 ± 562.4	0.250
HF power (msec^2^)	519.7 ± 371.0**	416.6 ± 296.6**	0.010
VLF power (msec^2^)	1684.6 ± 875.7	1623.1 ± 1006.5	0.448
LF/HF	1.4 ± 0.3**	1.5 ± 0.3**	0.004
SDNN (msec)	177.5 ± 29.9	173.2 ± 40.1	0.467
SDANN (msec)	160.7 ± 28.9	156.0 ± 37.8	0.421
rMSSD (msec)	49.0 ± 13.3	44.7 ± 14.8	0.062
pNN50 (%)	24.0 ± 9.9*	20.5 ± 10.5*	0.029
Circulating biomarkers
Fibrinogen (μg/mL)	2.5 ± 1.3	2.5 ± 1.3	0.801
P-selectin (ng/mL)	164.0 ± 2.0	200.3 ± 1.5	0.185
VCAM-1 (ng/mL)	1480.3 ± 1.5	1808.0 ± 1.6	0.138
ET-1 (pg/mL)	109.9 ± 1.6	121.5 ± 1.6	0.172
vWF (μg/mL)	24.5 ± 1.3	27.1 ± 1.3	0.337
Notes: DBP, diastolic blood pressure; ET-1, endothelin-1; HF, high frequency; LF, low frequency; LF/HF, the ratio of LF to HF; pNN50, percentage of normal RR intervals with duration > 50 msec different from the previous normal RR interval; rMSSD, the root mean square of the successive differences; SBP, systolic blood pressure; SDANN, the standard deviation of the average NN intervals calculated over short periods; SDNN, the standard deviation of the normal-to-normal interval; VCAM-1, vascular cell adhesion molecule-1; VLF, very low frequency; vWF, von Willebrand factor. *p < 0.05; **p < 0.01.			

**Figure 2:**
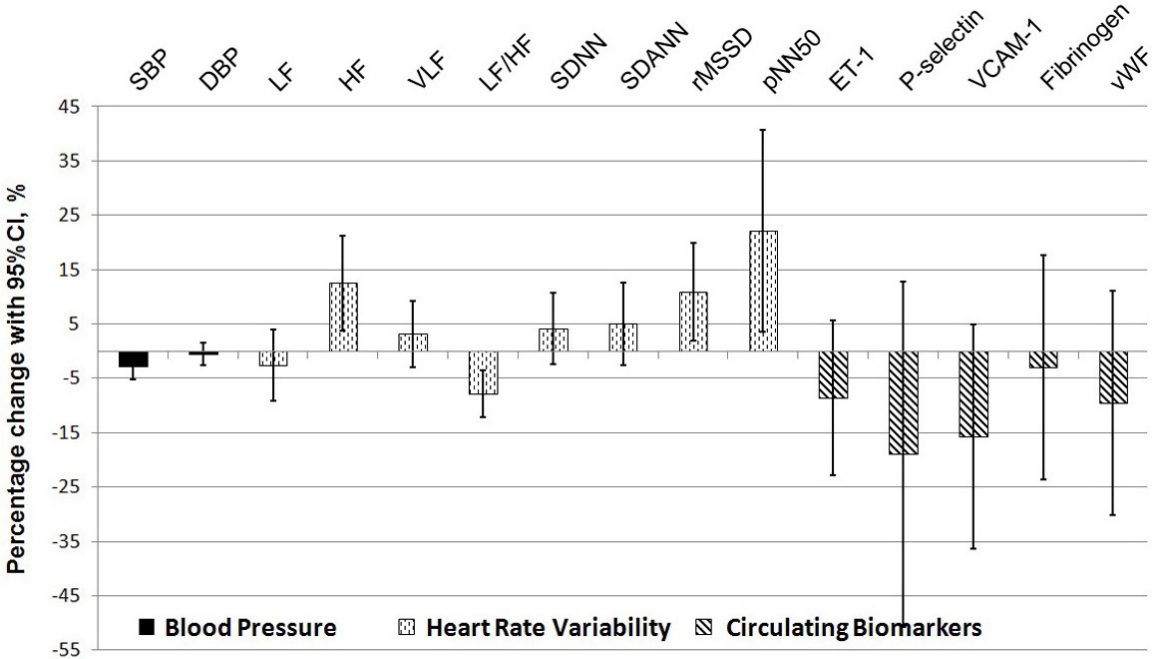
Percent change for various cardiovascular outcomes in the presence and absence of respirators. Notes: CI, confidence interval; DBP, diastolic blood pressure; ET-1, endothelin-1; HF, high frequency; LF, low frequency; LF/HF, the ratio of LF to HF; pNN50, percentage of normal RR intervals with duration > 50 msec different from the previous normal RR interval; rMSSD, the root mean square of the successive differences; SBP, systolic blood pressure; SDANN, the standard deviation of the average NN intervals calculated over short periods; SDNN, the standard deviation of the normal-to-normal interval; VCAM-1, vascular cell adhesion molecule-1; VLF, very low frequency; vWF, von Willebrand factor.

The mixed-effect linear model showed that subjects wearing respirators had a decrease of 2.7 mmHg (95% CI: 0.1, 5.2 mmHg) in systolic BP compared with those not wearing respirators. In the same comparison, there were increases of 12.5% (95% CI: 3.8%, 21.2%) in HF power, 3.2% (95% CI: –2.9%, 9.3%) in VLF power, 4.1% (95% CI: –2.4%, 10.7%) in SDNN, 10.9% (95% CI: 1.8%, 20.0%) in rMSSD, and 22.1% (95% CI: 3.6%, 40.7%) in pNN50% (Table 3, Figure 2). The use of respirators was also associated with decreases of 2.6% (95% CI: –3.9%, 9.1%) in LF power and 7.8% (95% CI: 3.5%, 12.1%) in LF/HF.

**Table 3:** Percent change (mean%, 95% CI) or differences (for BP) in various cardiovascular outcomes comparing the presence and absence of respirators.

Parameters	Change, % (95% CI)	*p*-Value
Blood pressure^*a*^
SBP (mmHg)	–2.7 (–5.2, –0.1)*	0.049
DBP (mmHg)	–0.5 (–2.5, 1.5)	0.622
Heart rate variability
LF power (msec^2^)	–2.6 (–9.1, 3.9)	0.262
HF power (msec^2^)	12.5 (3.8, 21.2)*	0.012
VLF power (msec^2^)	3.2 (–2.9, 9.3)	0.313
LF/HF	–7.8 (–12.1, –3.5)*	0.001
SDNN (msec)	4.1 (–2.4, 10.7)	0.231
SDANN (msec)	5.0 (–2.5, 12.6)	0.207
rMSSD (msec)	10.9 (1.8, 20.0)*	0.032
pNN50 (%)	22.1 (3.6, 40.7)*	0.037
Circulating biomarkers
Fibrinogen (μg/mL)	–3.0 (–23.6, 17.7)	0.765
P-selectin (ng/mL)	–18.9 (–50.7, 12.8)	0.192
VCAM-1 (ng/mL)	–15.6 (–36.2, 5.0)	0.122
ET-1 (pg/mL)	–8.6 (–22.8, 5.6)	0.168
vWF (μg/mL)	–9.5 (–30.1, 11.1)	0.331
Notes: BP, blood pressure; CI, confidence interval; DBP, diastolic blood pressure; ET-1, endothelin-1; HF, high frequency; LF, low frequency; LF/HF, the ratio of LF to HF; pNN50, percentage of normal RR intervals with duration > 50 msec different from the previous normal RR interval; rMSSD, the root mean square of the successive differences; SBP, systolic blood pressure; SDANN, the standard deviation of the average NN intervals calculated over short periods; SDNN, the standard deviation of the normal-to-normal interval; VCAM-1, vascular cell adhesion molecule-1; VLF, very low frequency; vWF, von Willebrand factor. Linear mixed-effect models were used to investigate the effects of wearing respirators, with adjustments for age, sex, body mass index, PM_2.5_ concentration, 48-hr mean temperature, and 48-hr mean humidity. ^***a***^The estimated effects for BP were the absolute difference in mmHg comparing the presence and absence of respirators. *p < 0.05.		

Wearing respirators was associated with decreases in several circulating biomarkers of inflammation [3.0% (95% CI: –23.6%, 17.7%) for fibrinogen, 18.9% (95% CI: –50.7%, 12.8%) for P-selectin, and 15.6% (95% CI: –36.2%, 5.0%) for VCAM-1], vasoconstriction [8.6% (95% CI: –22.8%, 5.6%) for ET-1] and blood coagulation [9.5% (95% CI: –30.1%, 11.1%) for vWF], but none of the associations was statistically significant.

## Discussion

With its randomized controlled crossover design, this intervention study demonstrated that wearing high-efficiency particulate-filtering respirators for a short time may improve HRV parameters and decrease BP levels in healthy young adults in Shanghai, China. This study is one of the few investigations to evaluate the health benefits of wearing respirators under urban air pollution levels in a developing country.

Consistent with the findings of previous studies, we found that a short-term exposure to ambient PM was associated with altered HRV (Dockery 2001). For example, a study of 120 healthy young subjects in Taiwan reported significant decreases in various HRV parameters associated with increased levels of PM_2.5_ (Liu et al. 2015). A recent meta-analysis of 18,667 participants enrolled in 29 studies reported an inverse relationship between parameters of HRV and exposure to PM (Pieters et al. 2012). Experimental studies have also supported the hypothesis that the autonomic nervous system is a potential target for the adverse effects of air pollution (Stone and Godleski 1999). Reduced HRV represents a withdrawal of cardiac vagal tone or an increase in sympathetic tone and is a predictor of poor prognosis in patients recovering from myocardial infarction or cardiac failure. Decreased HRV may further elevate the risk of cardiac arrhythmias in these at-risk patients (Odemuyiwa et al. 1991). These prior studies consistently revealed plausible pathways by which PM_2.5_ exposure affected the cardiovascular system.

In the present study, we explored the effects of PM_2.5_ on HRV in a group of healthy young subjects by reducing PM_2.5_ exposure through wearing high-efficiency respirators. A variety of mechanistic hypotheses by which inhaled particles may affect neural control of the heart involve both sympathetic and parasympathetic portions of the autonomic nervous system (Stone and Godleski 1999). When examining both time-domain and frequency-domain HRV parameters, the changes in HRV were predominantly observed in the HF power band, which is mainly influenced by parasympathetic tone. This finding indicates an increased contribution of parasympathetic tone to the heart autonomic function control when particulate exposure was reduced by the use of particulate-filtering respirators. In addition, the significant increase in pNN50 that occurred when wearing the respirator suggests increased parasympathetic cardiac autonomic control in response to lower PM_2.5_ exposure. In contrast, inhaled particles may promote a systemic sympathetic stress response that leads to decreased HRV and an increased ratio of LF/HF power (Stone and Godleski 1999).

Furthermore, we observed that a decrease in PM_2.5_ exposure was associated with lower BP levels, which was consistent with many previous findings (Brook et al. 2002; Pieters et al. 2012; Tsai et al. 2012). A panel study in Taiwan based on personal PM_2.5_ measurements and ambulatory BP monitoring showed that a 10 μg/m^3^ increase in PM_2.5_ was associated with significant increases in SBP by 0.81 mmHg (95% CI: 0.19, 1.43 mmHg) and in DBP by 0.63 mmHg (95% CI: 0.17, 1.10 mmHg) (Chang et al. 2015). In our study, we did not observe a significant effect on DBP, which was also consistent with the findings of some previous studies (Pieters et al. 2015; Zhang et al. 2013).

Blood inflammation, coagulation and vasoconstriction have been proposed to be mainly responsible for the biological mechanisms of the cardiovascular effects of PM_2.5_ (Chen et al. 2015a). We observed that wearing respirators led to lower levels of five circulating biomarkers of inflammation, coagulation, and vasoconstriction, although the decreases were not statistically significant. The weak effects on circulating biomarkers may be due to the relatively small sample size, the short intervention period, the relatively young age of the participants, and the incomplete control of the exposure scenarios, given that 10% of the participants did not wear respirators for ~ 20% of the time. We may expect larger effects with longer intervention periods and in older or susceptible population subgroups.

The evaluation of individual intervention approaches in reducing hazards associated with PM_2.5_ is crucial to public health. We found that a short-term use of respirators might result in an improvement in cardiac autonomic nervous function and a decrease in BP. The results for HRV and BP were consistent with the findings from two previous intervention studies using respirators in healthy volunteers and patients with coronary heart disease (Langrish et al. 2009, 2012) in Beijing. Considering these cardiovascular health parameters together, there might be complementary roles among them. For example, the decreased inflammatory levels might cause increased HRV and further lower BP, or increased HRV might aid in decreasing BP. However, we did not measure or collect the samples at different time lags; this suggests a possible direction for further research.

There are several limitations in this study. First, when wearing respirators, participants might feel increased respiration resistance, which might have increased their anxiety. This increased anxiety might in turn trigger sympathetic nervous system tone and hence lead to an increase in LF power. A double-blind design using a sham face-piece respirator would have helped remove the effect of anxiety. However, we did not have sufficient resources to find sham respirators that exactly resembled the experimental respirators. Second, we recruited healthy college students rather than those more susceptible to PM (such as patients with chronic cardiovascular diseases) to better control for potential confounding that might have been difficult to control in other study settings (e.g., indoor cooking, smoking, medication use, and individual health status). Therefore, caution should be used when extrapolating our findings to other subgroups. Third, the short-term nature of this study might have led us to underestimate or miss some potential lagged health benefits attributable to respirators. Fourth, our findings were exploratory in nature because of the relatively small sample size. Fifth, exposure measurement errors were inevitable because PM_2.5_ was not measured at the individual level. The monitoring devices were not calibrated by gravimetric measurements, and some indoor sources (e.g., human activities) were likely to influence the participants’ exposure to PM_2.5_. Sixth, we did not measure physical activity level directly, which might confound our results to some extent. Therefore, larger studies on long-term use of respirators in vulnerable populations are needed to confirm our findings.

## Conclusion

In summary, this randomized crossover study suggested that short-term wearing of particulate-filtering respirators may produce cardiovascular benefits in improving autonomic nervous function and lowering BP levels. Our findings provide preliminary evidence that a respirator may serve as an effective and practical tool to protect individual cardiovascular health from particulate air pollution in a developing country with severe air pollution problems.
